# Association of Sodium-Glucose Cotransporter 2 Inhibitors With Cardiovascular Outcomes in Patients With Type 2 Diabetes and Other Risk Factors for Cardiovascular Disease: A Meta-analysis

**DOI:** 10.1001/jamanetworkopen.2021.42078

**Published:** 2022-01-05

**Authors:** Mukul Bhattarai, Mohsin Salih, Manjari Regmi, Mohammad Al-Akchar, Radhika Deshpande, Zurain Niaz, Abhishek Kulkarni, Momin Siddique, Shruti Hegde

**Affiliations:** 1 Division of Cardiology, Department of Internal Medicine, Southern Illinois University School of Medicine, Springfield; 2 Department of Internal Medicine, Southern Illinois University School of Medicine, Springfield

## Abstract

**Importance:**

The cardiovascular outcome in selected populations when sodium-glucose cotransporter 2 inhibitors (SGLT2-Is) are emerging as standard therapy is not clearly understood. It is important to learn the magnitude of cardiovascular benefit using SGLT2-Is across the select subgroups that include both sexes and multiple age and racial and ethnic groups.

**Objectives:**

To evaluate the association between use of SGLT2-Is and cardiovascular benefits in a prespecified group in a larger sample size using data obtained from randomized clinical trials.

**Data Sources:**

Search of electronic databases PubMed, Google Scholar, Web of Science, and Cochrane from inception to January 10, 2021, with additional studies identified through conference papers and meeting presentations, ClinicalTrials.gov, and reference lists of published studies.

**Study Selection:**

Placebo-controlled randomized clinical trials in which participants had atherosclerotic cardiovascular disease (ASCVD) or risk factors for ASCVD, diabetes, or heart failure and which reported the primary outcome were included in this study. Multicenter observational and nonobservational studies and those with different outcomes of interest were excluded.

**Data Extraction and Synthesis:**

Medical Subject Heading search terms included *SGLT2-I* and multiple cardiovascular outcomes in different combinations. The study followed the Preferred Reporting Items for Systematic Reviews and Meta-analyses (PRISMA) reporting guideline. The analysis of all outcomes was performed using a Mantel-Haenszel equation and the random-effects model.

**Main Outcomes and Measures:**

Six efficacy outcomes of SGLT2-I use (cardiovascular death and hospitalization for heart failure [HHF] as the primary outcome and major adverse cardiovascular event, HHF, cardiovascular death, acute myocardial infarction, and all-cause mortality as secondary outcomes), were evaluated. Subgroup analysis was performed for the primary outcome of cardiovascular death or HHF. Odds ratios (ORs) and 95% CIs were used to compare 2 interventions.

**Results:**

Ten studies with 71 553 participants were included, among whom 39 053 received SGLT2-Is; among studies that reported these data, 28 809 were men and 15 655 were women (mean age, 65.2 [range, 61.9-70.0] years). Race and ethnicity were defined in the original trials and were categorized as Asian, Black, or other (6900 participants) and White (26 646 participants) for the purposes of this analysis (the category “other” was not specified consistently). In terms of age, 16 793 were younger than 65 years and 17 087 were 65 years or older. At a mean follow-up 2.3 (range, 0.8-4.2) years, the SGLT2-I group favored reduction in primary outcome (3165 of 39 053 [8.10%] vs 3756 of 32 500 [11.56%]; OR, 0.67 [95% CI, 0.55-0.80]; *P* < .001). No difference was noted in the rate of acute myocardial infarction compared with the placebo group (1256 of 26 931 [4.66%] vs 958 of 20 373 [4.70%]; OR, 0.95 [95% CI, 0.87-1.03]; *P* = .22). Subgroup analysis favored SGLT2-I use for the primary outcome in both sexes, age groups, and racial and ethnic groups.

**Conclusions and Relevance:**

This meta-analysis supports that SGLT2-Is have emerged as an effective class of drugs for improving cardiovascular morbidity and mortality in selected patients. Sodium-glucose cotransporter 2 inhibitors were not associated with reduced risk of acute myocardial infarction. Future long-term prospective studies are warranted to understand the long-term cardiovascular benefits.

## Key Points

**Question:**

What is the updated magnitude of benefit associated with sodium-glucose cotransporter 2 inhibitors (SGLT2-Is) on outcome of cardiovascular death or hospitalization for heart failure (HHF) in different select subgroups of patients?

**Findings:**

This meta-analysis of 10 high-quality randomized clinical trials (71 553 participants) found that use of SGLT2-Is was associated with lower occurrence of cardiovascular death or HHF by 33% in high-risk patients. For the primary outcome of HHF or cardiovascular death, the study showed equal benefit of SGLT2-Is in both sexes; lower risk in participants who were younger than 65 years compared with those 65 years or older; and greater risk reduction in participants who were Asian, Black, or of other races or ethnicities compared with White participants.

**Meaning:**

These findings suggest that SGLT2-Is may be associated with an overall cardiovascular benefit for patients with high-risk cardiovascular features compared with placebo in both sexes, different age groups and different races and ethnicities.

## Teaser

This meta-analysis assesses whether use of sodium-glucose cotransporter 2 inhibitors (SFLT2-Is) is associated with cardiovascular benefits in patients with atherosclerotic cardiovascular disease or its risk factors, diabetes, or heart failure

## Introduction

Sodium-glucose cotransporter 2 inhibitors (SGLT2-Is), also known as a glifozins, constitute a class of medication that was initially approved as an antidiabetic agent because the mechanism of action consisted of lowering blood glucose levels by promoting excretion of glucose through the kidneys via renal tubules.^1,2^ As the medication was further studied, it was found to have positive cardioprotective effects in multiple recent large-scale randomized clinical trials (RCTs), not limited to the patients with type 2 diabetes. The CREDENCE (Effects of Canagliflozin on Renal and Cardiovascular Outcomes in Participants With Diabetic Nephropathy),^3^ SCORED (Sotagliflozin in Patients with Diabetes and Chronic Kidney Disease),^4^ CANVAS (Canagliflozin Cardiovascular Assessment Study),^5^ EMPA-REG OUTCOME (Empagliflozin Cardiovascular Outcome Event Trial in Type 2 Diabetes Mellitus Patients),^6^ DECLARE-TIMI 58 (Dapagliflozin and Cardiovascular Outcomes in Type 2 Diabetes),^7^ VERTIS-CV (Cardiovascular Outcomes With Ertugliflozin in Type 2 Diabetes),^8^ and SOLOIST-WHF (Sotagliflozin in Patients With Diabetes and Recent Worsening Heart Failure)^9^ trials evaluated cardiovascular benefits of SGLT2-I use in patients with diabetes. The DAPA-HF (Dapagliflozin in Patients with Heart Failure and Reduced Ejection Fraction),^10^ DAPA-CKD (Dapagliflozin in Patients with Chronic Kidney Disease),^11^ and EMPEROR-Reduced (Cardiovascular and Renal Outcomes with Empagliflozin in Heart Failure)^12^ trials enrolled both patients with and without diabetes. Cardiovascular benefits in patients with heart failure regardless of the presence of diabetes in the DAPA-HF, EMPEROR-Reduced, and SOLOIST-WHF trials included only heart failure among patients with reduced ejection fraction.^3,5,6^ The VERTIS-CV,^8^ EMPA-REG OUTCOME,^6^ SCORED,^4^ DECLARE TIMI,^7^ and CANVAS^5^ trials included patients with established cardiovascular disease (CVD) or at least 1 risk factor for CVD. These trials compared cardiovascular outcomes, with each trial focusing on different risk factors in the population. Meanwhile, the new VERTIS-CV^8^ trial showed that the outcome of death from cardiovascular causes and hospitalization for heart failure (HHF) did not differ between the SGLT2-I group and the placebo group. A recently published trial, SOLOIST-WHF,^9^ studied the cardiovascular outcomes in patients with worsening of existing heart failure requiring hospital treatment. All RCTs found significant reductions in cardiovascular death and HHF in patients receiving SGLT2-Is except CANVAS,^5^ which found a reduction in HHF but not in cardiovascular death. All these studies showed a variable degree of benefit in the SGLT2-I group compared with the placebo group for cardiovascular death or HHF.

Our meta-analysis aimed to interpret various outcomes from these studies and compare them with one another. This meta-analysis is taken from large RCTs within the last 7 years. Furthermore, this meta-analysis ensures the generalizability of data from all these trials because patients with and without diabetes, with and without heart failure, and with cardiovascular risk factors were studied and analyzed altogether. In contrast, prior meta-analyses by Butler et al^13^ and Docherty et al^14^ primarily studied patients with heart failure and their cardiovascular outcomes, whereas our study included patients without heart failure and accounted for the presence of cardiovascular risk factors. Another meta-analysis by Zelniker et al^15^ is similar to our meta-analysis but was performed in 2019 and hence did not include the emerging data from our study. Furthermore, the present meta-analysis is the first large study, to our knowledge, that has also focused on cardiovascular outcomes in both sexes and different age and racial and ethnic groups to understand the magnitude of benefit across the subgroups, because this is essential to initiate SGLT2-I use as standard therapy.

## Methods

### Selection of Studies

**Figure 1.**
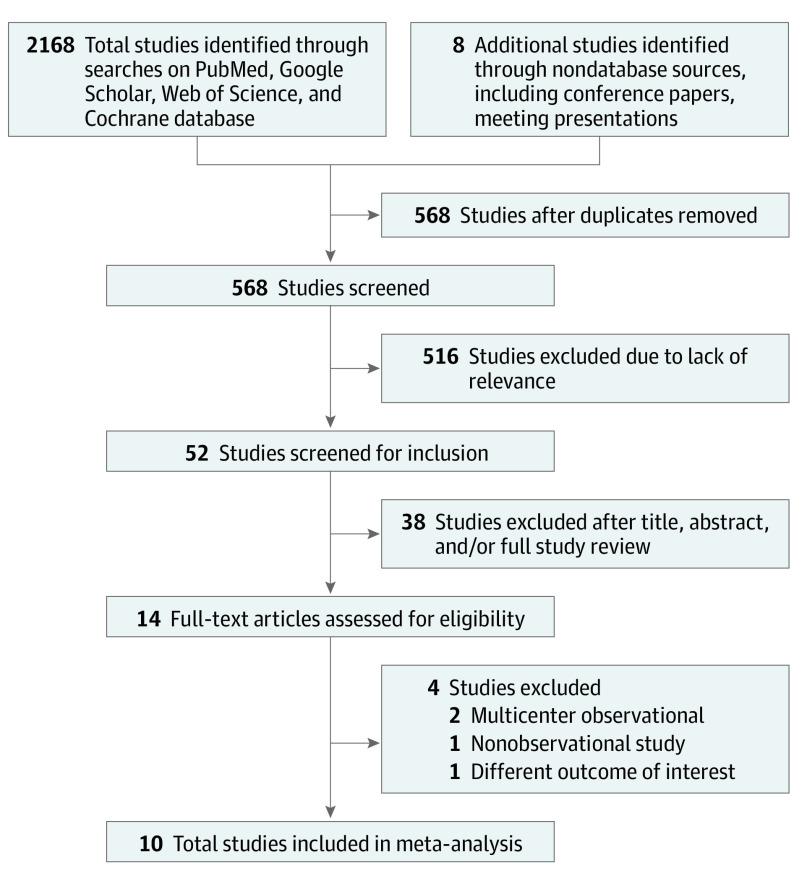
PRISMA Flow Diagram of Study Selection

Two authors (M.B and M.R.R) separately explored Google Scholar, PubMed, Web of Science, and Cochrane from database inception to January 10, 2021. The search was further expanded to conference proceedings, ClinicalTrials.gov, and reference lists of published trials, reviews, and meta-analyses. Keyword and Medical Subject Heading search terms included *SGLT2-I* and *cardiovascular outcomes*, *dapagliflozin* and *cardiovascular outcomes*, *canagliflozin* and *cardiovascular outcomes*, *empagliflozin* and *cardiovascular outcomes*, and *ertugliflozin* and *cardiovascular outcomes*. The search terms for cardiovascular outcomes included *major adverse cardiovascular event (MACE)*, *cardiovascular death*, *cardiovascular readmission*, *heart failure*, *acute myocardial infarction (MI)*, and *all-cause mortality*. In addition, the different combinations of these terms were used in each database search. Final eligible studies were selected with the consensus of all authors. The study followed the Preferred Reporting Items for Systematic Reviews and Meta-analyses (PRISMA) reporting guideline providing the search strategy to obtain all eligible studies. The PRISMA flow diagram and the reasons for study exclusion are provided in Figure 1.

The following standard criteria were set to select eligible RCTs comparing outcomes of SGLT2-I use vs a placebo control group. The baseline characteristics included established atherosclerotic cardiovascular disease (ASCVD), a high risk of ASCVD, or heart failure with or without kidney disease and diabetes; in addition, the trial reported our primary outcome, which is cardiovascular death or HHF. After removing the duplicates, a total of 568 studies were screened. A total of 52 studies were selected to screen abstracts with or without a full study review. Ten high-quality studies were eligible for the meta-analysis. One RCT^16^ did not match our outcomes of the cardiovascular benefit because its outcome was an improvement in left ventricular systolic function. Multiple observational studies^17,18^ were excluded from the meta-analysis. Several trials that have not yet released their final results were not included in the study. Ten placebo-controlled RCTs met the inclusion criteria.^3,4,5,6,7,8,9,10,11,12^ Any discrepancies in data extraction or risk-of-bias assessment were resolved with the consensus of all authors.

### Quality Assessment

The modified Jadad Score was used to assess the methodological quality of RCTs. A score of 0 to 8 quantified the quality of each trial; high-quality studies (score ≥3) were included, as shown in eTable 1 in the Supplement.

### Outcomes

The primary efficacy outcomes were cardiovascular death or HHF. The secondary outcomes were MACE, HHF, cardiovascular death, acute MI, and all-cause mortality. MACE was defined as death due to cardiovascular causes, nonfatal MI, or nonfatal stroke. Both safety and efficacy outcomes were analyzed by performing a meta-analysis. The prespecified subgroup analyses were performed for sex (men vs women), age groups (<65 or ≥65 years), and racial and ethnic groups, the latter of which were defined in the original trials and were categorized as Asian, Black, or other race or ethnicity (in which the category “other” was not specified consistently) or White for the purposes of our analyses. In the selected population the benefit of SGLT2-I is not clearly understood when SGLT2-Is are emerging as standard therapy in this current era. The data was abstracted by observing event rates in different race and ethnicity groups from each included trial. The exclusion and inclusion criteria of each study, including primary and secondary outcomes, are all presented separately (eTable 2 in the Supplement)

### Data Analysis

Cochrane Review Manager (RevMan) software, version 5.3 (The Nordic Cochrane Center) was used to perform pairwise analysis. The data from studied trials were used to calculate odds ratios (ORs) and 95% CIs comparing the intervention vs the placebo groups. The analysis of all outcomes was performed using a Mantel-Haenszel equation and the random-effects model. A 2-sided *P* < .05 was considered statistically significant for all analyses. Heterogeneity was tested using *I*^2^ and χ^2^ tests. *I*^2^ index values of 25% to 50% were considered low heterogeneity; 51% to 75%, moderate heterogeneity; and greater than 75%, high heterogeneity. Publication bias was evaluated by visual inspection of the funnel plot (eFigure 1 in the Supplement).

## Results

A total of 10 trials involving 71 553 patients in a high-risk cardiac disease study group were included in the analysis.^3,4,5,6,7,8,9,10,11,12^ A total of 39 053 patients received SGLT2-Is and 32 500 received placebo. Among patients with data available, 28 809 were men and 15 655 were women. Further, 26 646 White patients (79.43%) were included compared with 6900 patients (25.57%) who were Asian (4589 [66.51%]), Black (1321 [19.14%]), or of other race or ethnicity (990 [14.35%]). There were 16 793 participants who were younger than 65 years and 17 087 who were 65 years or older. Patients who received SGLT2-Is were compared with the patients who received a placebo (Table). All the patients had established ASCVD risk or cardiovascular risk factors. A total of 6 primary and secondary outcomes were evaluated. The mean follow-up was 2.3 (range, 0.8-4.2) years.

**Table.** Salient Features of Study and Participants of Included Placebo-Controlled RCTs

Source	Type of SGLT2-I (dose, mg)	No. of participants		Mean follow-up, y	Age, mean (SD), y	Sex, %		Established CVD, %	History of CHF, No. (%)	Mean LVEF, %	Reduced eGFR,^a^ No. of patients	Mean (SD)	
		SGLT2-I group	Placebo group			Women	Men					HgA_1c_ level, %	BMI
Zinman et al,^6^ 2015	Empagliflozin (10 and 25)	2333	4687	3.1	63 (8.7)	28.5	71.5	7020 (100)	706 (10.1)	27.5	1819	8.1 (0.8)	30.6 (5.3)
Neal et al,^8^ 2017	Canagliflozin (100 and 300)	5795	4347	2.4	63.3 (8.3)	35.8	64.2	6656 (65.6)	1461 (14.4)	NA	2039	8.2 (0.9)	32 (6)
Perkovic et al,^3^ 2019	Canagliflozin (100)	2202	2199	2.62	63.0 (9.2)	33.9	66.1	2223 (50.5)	652 (14.8)	NA	2592	8.3 (1.3)	31.3 (6.2)
McMurray et al,^10^ 2019	Dapagliflozin (10)	2373	2371	1.52	66.3 (10.9)	23.4	76.6	4744 (100)	4744 (100)	31	1226	NA	28.2 (6)
Wiviott et al,^9^ 2019	Dapagliflozin (10)	8582	8578	4.2	63.9 (6.8)	37.4	62.6	6974 (40.6)	1724 (10.0)	NA	1265	8.3 (1.2)	32 (6)
Heerspink et al,^11^ 2020	Dapagliflozin (10)	2152	2152	2.4	61.85 (12.1)	33.1	66.9	1610 (37.4)	468 (10.9)	NA	3850	NA	29.5 (6.2)
Packer et al,^12^ 2020	Empagliflozin (10)	1863	1867	1.33	66.85 (11)	23.9	76.1	3730 (100)	3730 (100)	NA	1799	NA	27.9 (5.4)
Cannon et al,^8^ 2020	Ertugliflozin (5 and 15)	5499	2747	3.5	64.4 (8.05)	29.9	70.1	8238 (99.9)	1958 (23.7)	NA	1807	8.2 (0.95)	31.95 (5.5)
Bhatt et al,^9^ 2021	Sotagliflozin (200 to >400)	608	614	0.75	70 (NA)	33.7	66.3	1222 (100)	1222 (100)	35	Mean 49.7	7.1 (NA)	30.8 (NA)
Bhatt et al,^4^ 2021	Sotagliflozin (200 to >400)	5292	5292	1.33	69 (NA)	44.9	55.1	NA	3283 (31.0)	NA	NA	8.3 (NA)	31.8 (NA)

Abbreviations: BMI, body mass index (calculated as weight in kilograms divided by height in meters squared); CHF, congestive heart failure; CVD, cardiovascular disease; eGFR, estimated glomerular filtration rate; HgA_1c_, hemoglobin A_1c_; NA, not applicable; RCT, randomized clinical trial; SGLT2-I, sodium-glucose cotransporter 2 inhibitor.

^a^ Indicates less than 60 mL/min/1.73 m^2^.

The primary outcome of cardiovascular death or HHF was reported in all 10 studied trials, with 6921 incidences among 71 553 patients. The primary outcome of cardiovascular death or HHF was significantly different from that of the placebo group (3165 of 39 053 [8.10%] vs 3756 of 32 500 [11.56%]; OR, 0.67 [95% CI, 0.55-0.80]; *P* < .001; *I^2^* = 92%) (eFigure 2 in the Supplement), with both outcomes being lower in the SGLT2-I group compared with the placebo group. SGLT2-I use was associated with decreased risk of cardiovascular death or HHF by 33%, with a number needed to treat of 5.7 at *P* < .001, and there was a 2.44% decreased event rate in the SGLT2-I group compared with the placebo group. Moreover, patients receiving SGLT2-Is were also found to have a statistically significant difference in MACE, with the SGLT2-I group having decreased MACE outcomes compared with the placebo group (3149 of 32 057 [9.82%] vs 2606 of 25 496 [10.22%]; OR, 0.90 [95% CI, 0.81-0.99]; *P* = .03; *I*^2^ = 66%) (eFigure 3 in the Supplement).

The SGLT2-I group had a decreased rate of HHF and emergency department visits with heart failure (1612 of 36 901 [4.37%] vs 2068 of 30 348 [6.81%]; OR, 0.67 [95% CI, 0.62-0.72]; *P* < .001; *I*^2^ = 8%) (eFigure 4 in the Supplement) and cardiovascular death (1815 of 39 053 [4.65%] vs 1672 of 32 500 [5.14%]; OR, 0.87 [95% CI, 0.79-0.97]; *P* = .009; *I*^2^ = 52%) (eFigure 5 in the Supplement) when compared with the placebo group. The cardiovascular death outcome was evaluated in all 10 studies.

Five studies^5,6,7,8,10^ evaluated acute MI and demonstrated no difference in outcome due to SGLT2-I therapy (1256 of 26 931 [4.66%] vs 958 of 20 373 [4.70%]; OR, 0.95 [95% CI, 0.87-1.03]; *P* = .22) when compared with placebo (eFigure 6 in the Supplement). No significant heterogeneity was noted in this event rate (*I*^2^ = 0%). All-cause mortality was obtained from all trials and was found to be significantly lower in the SGLT2-I group compared with the placebo group (2767 of 39 053 [7.09%] vs 2556 of 32 500 [7.86%]; OR, 0.87 [95% CI, 0.80-0.96]; *P* = .004; *I*^2^ = 59%) (eFigure 7 in the Supplement).

**Figure 2.**
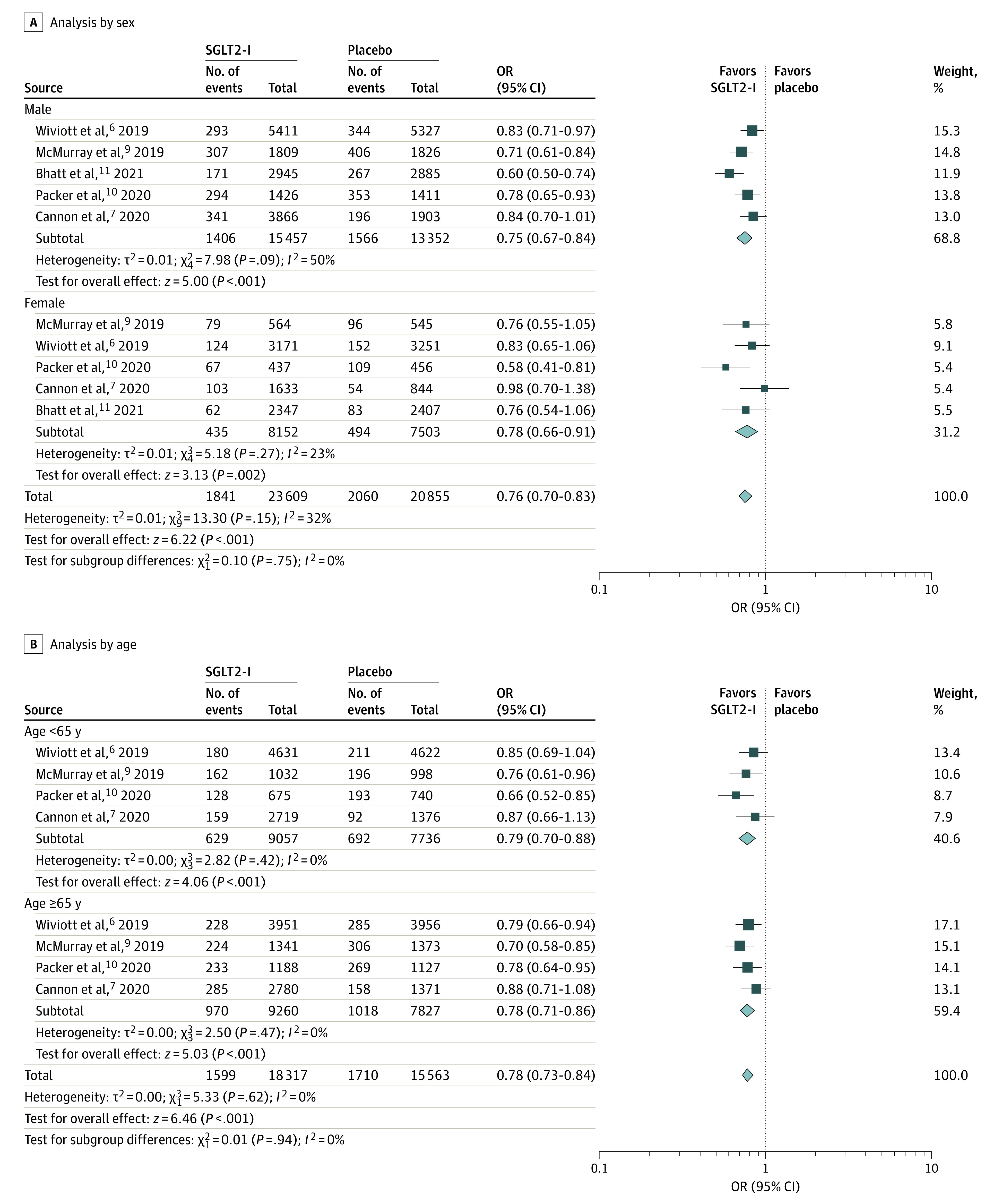
Subgroup Analysis by Sex and Age Odds ratios (ORs) were calculated using a Mantel-Haenszel equation with a random-effects model. SGLT2-I indicates sodium-glucose cotransporter 2 inhibitors.

**Figure 3.**
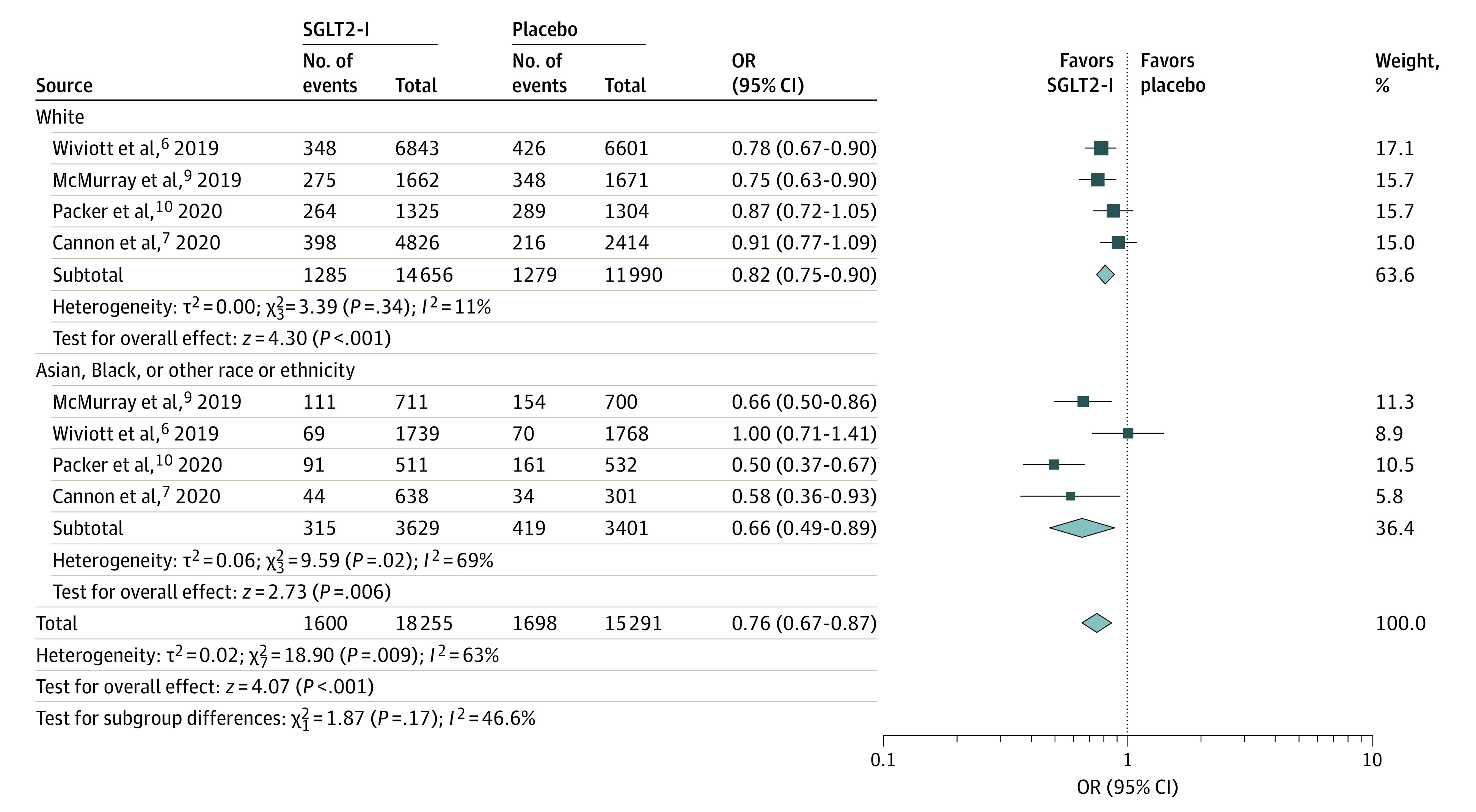
Subgroup Analysis by Race Odds ratios (ORs) were calculated using a Mantel-Haenszel equation with a random-effects model. SGLT2-I indicates sodium-glucose cotransporter 2 inhibitors.

Further subgroups analyses of the primary outcome were performed based on sex and age (Figure 2) and race and ethnicity (Figure 3). There was no difference in the outcome of the SGLT2-I group in men and women when compared with the placebo group. Pooled data from 5 studies^4,7,8,10,12^ showed that the rate of cardiovascular death or HHF in men (1406 of 15 457 [9.01%]; OR, 0.75 [95% CI, 0.67-0.84]; *P* < .001) was slightly higher than that in women (1841 of 23 609 [5.34%]; OR, 0.78 [95% CI, 0.66-0.91]; *P* = .002). We further divided participants between those younger than 65 years and 65 years or older. Both age groups favored SGLT2-I use, although the occurrence of primary outcome was slightly lower in those younger than 65 years (629 of 9057 [6.94%]; OR, 0.79 [95% CI, 0.70-0.88]; *P* < .001) when compared with those 65-years or older (970 of 9260 [10.47%]; OR, 0.78 [95% CI, 0.71-0.86]; *P* < .001). Heterogeneity was at 0% in all subgroups. Similarly, both White groups (1285 of 14 656 [8.77%]; OR, 0.82 [95% CI, 0.75-0.89]; *P* < .001) and groups who were Asian, Black, or of other race or ethnicity (315 of 3599 [8.75%]; OR, 0.66 [95% CI, 0.49-0.89]; *P* = .006) had favorable results with SGLT2-Is.

## Discussion

This meta-analysis presents the comprehensive pooled data from 10 contemporary large, placebo-controlled RCTs. The major findings include the following. First, cardiovascular death or HHF was decreased in the SGLT2-I group compared with the placebo group. Second, all-cause mortality was decreased in the SGLT2-I group compared with the placebo group. Third, MACE outcomes were decreased in the SGLT2-I group compared with the placebo group. Fourth, the SGLT2-I group had a decreased rate of HHF or emergency department visits for heart failure compared with the placebo group. Fifth, there was no difference in outcome for acute MI between the SGLT2-I and placebo groups.

Our study found that SGLT2-I use was associated with decreased risk of cardiovascular death or HHF by 33%, with a number needed to treat of 5.7 at *P* < .001. This result was significant when HHF admissions and cardiovascular death were analyzed independently as well. There was a 2.44% decreased event rate in the SGLT2-I group compared with the placebo group. These results are consistent with those reported by Butler et al^13^ in their meta-analysis that studied SGLT2-I use specifically for heart failure, whereas our study found these benefits in all patients with and without heart failure who are at high risk of CVD.

Our findings show that the SGLT2-I group had a statistically significant reduction in MACE outcomes compared with the placebo group (OR, 0.90 [95% CI, 0.81-0.99]). Two trials, SCORED^4^ and CREDENCE,^3^ contributed to the positive outcomes of this study. Both trials specifically studied patients with diabetes and chronic kidney disease and had strongly positive MACE outcomes. The VERTIS-CV,^8^ CANVAS,^5^ and DECLARE-TIMI 58^7^ trials all had borderline results that showed no significant difference between the SGLT2-I and placebo groups. There was no benefit in the CANVAS trial^5^ when the individual outcomes of cardiovascular death and nonfatal MI were studied. The VERTIS-CV^8^ and DECLARE-TIMI 58^7^ trials found that SGLT2-I treatment was noninferior compared with placebo in terms of MACE outcomes. A meta-analysis of all these studies suggests a benefit of SGLT2-Is over placebo. This finding is likely owing to the increased power of this meta-analysis compared with individual studies.

The all-cause mortality outcome was collected and evaluated from all trials. The combined result suggests a clear benefit associated with SGLT2-Is compared with placebo (*P* = .004). This finding suggests that there may be an overall benefit of SGLT2-Is for patients and vouches for the safety of the medication.

Five studies^5,6,7,8,10^ evaluated acute MI and individually showed no difference in outcomes. The combined results of these 5 studies demonstrated no difference in outcome between SGLT2-I therapy and placebo (*P* = .22). No significant heterogeneity was noted in this event rate (*I*^2^ = 0%).

Subgroup analyses of the primary outcome were performed based on sex, age, and race and ethnicity. Equal benefit of SGLT2-Is was observed in both sexes (OR for men, 0.75; OR for women, 0.78) for the primary outcome of cardiovascular death or HHF. Pooled data from 5 studies showed that the rate of cardiovascular death or HHF in men is slightly higher than that of women (9.01% vs 5.34%). There were more men in the study than women, and the greater rate of cardiovascular death or HHF in men could be attributed to the difference in the sample size.

For another subgroup analysis, participants were divided into those younger than 65 years and 65 years or older. Age is a significant independent risk factor for cardiovascular death.^19^ Heart failure is more common in elderly individuals and is more common in older women than in older men.^19^ Our findings suggest that SGLT2-Is effectively reduce the outcome of cardiovascular death or HHF in patients in both age groups with similar risk reduction. However, the cardiovascular death or HHF outcome was reduced in patients younger than 65 years when compared with those 65 years or older. This could imply a benefit of starting SGLT2-I therapy at an earlier age rather than an older age (>65 years) to prevent further advance of the disease. There is significant potential to reduce morbidity and mortality caused by CVD and provide substantial economic benefit to one of the leading causes of US health care expenditure.

Similarly, both White groups and groups who were Asian, Black, or of other race or ethnicity had a reduction in cardiovascular death or HHF in the SGLT2-I group compared with the placebo group (ORs, 0.82 and 0.66, respectively). However, the sample size for the groups who were Asian, Black, or of other race or ethnicity was smaller in all the studies (total, 6900 vs 26 646 for the White group). This finding is important because the rates of CVD and comorbidities were greater in the cohort of participants who were Asian, Black, or of other race or ethnicity, and SGLT2-I use confers benefits to both groups equally. There was a lack of heterogeneity in all subgroups in our study. With the findings from a subgroup analysis, use of SGLT2-Is as a novel therapeutic approach is suggested as an important measure to prevent cardiovascular death or HHF in both sexes, age groups, and racial and ethnic groups regardless of the presence of multiple comorbidities.

The study population in this meta-analysis included patients with heart failure and those at a high risk of ASCVD. We emphasize that at present more than 5.8 million patients have congestive heart failure in the US alone and more than 1 million are hospitalized per year as a result of heart failure. It has become the most common hospital admission diagnosis of patients covered by Medicare and is the leading cause of rehospitalization within 30 days.^20,21^ Among patients with heart failure 64 years and older, 23.2% are readmitted within 30 days. Each admission for heart failure incurs an estimated cost of $14 631, resulting in a substantial economic burden.^22^ The amalgamated pooled data from these trials in our study suggests an overall significant reduction in cardiovascular death or HHF, MACE outcomes, and all-cause mortality in patients treated with SGLT2-Is with every degree of comorbidity from low to high risk. The study population included only patients with congestive heart failure from high-quality trials, including DAPA-HF,^10^ VERTIS-CV,^8^ and SOLOIST.^9^ Five studies (EMPA-REG outcome,^6^ DAPA-HF,^10^ EMPEROR REDUCED,^12^ VERTIS-CV,^8^ and SOLOIST^9^) include only patients with established cardiovascular disease, and 4 studies (CANVAS,^5^ CREDENCE,^3^ DECLARE-TIMI,^7^ and DAPA-CKD^11^) include patients with prior ASCVD rates of 65.6%, 50.5%, 40.6%, and 37.4%, respectively.

The cardiac benefits of SGLT2-Is have been hypothesized to be due to several mechanisms. The cardioprotective effects of SGLT2-Is are secondary to an improvement in ventricular load through a reduction in preload by eliciting natriuresis and osmotic diuresis and a decrease in afterload by lowering blood pressure and improving vascular function.^23^ In addition, SGLT2-Is have been compared in other studies with loop diuretics; both were associated with a similar natriuretic effect and reduced interstitial fluid. These natriuretic effects may explain the findings in reducing HHF.^23^ In addition, SGLT2-Is are believed to improve cardiac metabolism by optimizing the use of ketones.^23^ Last, SGLT2-Is have been associated with the inhibition of cardiac fibrosis, which is considered an important heart failure pathway.^24^ These mechanisms of action will not be relevant in the pathophysiology of acute ischemia to the myocardium. Sodium-glucose cotransporter 2 inhibitors do not have known antianginal properties or vasodilatory effects, do not reduce myocardial oxygen consumption, and do not prevent remodeling of the cardiac muscle. This is most likely the reason why SGT2-I is not beneficial for acute MI. A previous study^25^ analyzing the broad spectrum of safety profiles of SGLT2-Is demonstrated a nonsignificant occurrence of major hypoglycemic events, acute kidney injury, fracture, bladder cancer, Fournier gangrene, amputation, and urinary tract infection but a slightly increased association of diabetic ketoacidosis.

### Limitations

There are several limitations to our study. First, we lacked access to patient-level data to perform a propensity analysis or stratified analysis, which could better define differences between treatment groups with regard to patient characteristics, clinical presentation, and procedural characteristics. The definition of secondary outcomes was variable across studies, which may affect the outcome assessment. We have limited the selection bias by excluding nonrandomized and observational studies. We have further reduced the bias to a large extent by analyzing all evidence available and independently extracting randomized data. The several outcomes of SGLT2-I therapy from the included studies may not represent definitive incidence because the occurrence pattern may vary in a larger population than in the initial trials.

## Conclusions

Our meta-analysis evaluated a wide spectrum of efficacy outcomes, further characterizing the primary outcome in different subgroups from several well-designed large clinical trials. It supports that SGLT2-Is have emerged as an effective class of drugs for improving cardiovascular morbidity and mortality, including the prevention of HHF and reducing all-cause mortality in selected patients. SGLT2-I therapy did not reduce the risk of acute MI. The cardiovascular outcomes of SGLT2-I therapy can be compared across all trials, and it demonstrates remarkable consistency of class benefit, despite the variations in populations enrolled. Owing to the short-term trial durations, future long-term prospective studies and postmarketing surveillance studies are warranted to discover the rate of cardiovascular outcomes.

## Notes

Supplement. **eTable 1.** Modified Jadad Scores of the Included Studies **eTable 2.** Definition of Inclusion, Exclusion, Primary Outcome, and Secondary Outcome **eFigure 1.** Funnel Plot **eFigure 2.** Analysis of Study Outcomes: Cardiovascular Death or Hospitalization for Heart Failure **eFigure 3.** Analysis of Study Outcomes: MACE **eFigure 4.** Analysis of Study Outcomes: Rates of HHF and Emergency Department Visits for Patients With Heart Failure **eFigure 5.** Analysis of Study Outcomes: Rates of HHF and Emergency Department Visits for Patients With Cardiovascular Death **eFigure 6.** Analysis of Study Outcomes: Acute Myocardial Infarction **eFigure 7.** Analysis of Study Outcomes: All-Cause Mortality
