# Supplementary material for: Association of Sodium-Glucose Cotransporter 2 Inhibitors With Cardiovascular Outcomes in Patients With Type 2 Diabetes and Other Risk Factors for Cardiovascular Disease: A Meta-analysis
Source: JAMA Netw Open. 2022 Jan 5;5(1):e2142078. doi: 10.1001/jamanetworkopen.2021.42078 (PMC8733833; doi:10.1001/jamanetworkopen.2021.42078)
Supplement: Supplement. — eTable 1. Modified Jadad Scores of the Included Studies eTable 2. Definition of Inclusion, Exclusion, Primary Outcome, and Secondary Outcome eFigure 1. Funnel Plot eFigure 2. Analysis of Study Outcomes: Cardiovascular Death or Hospitalization for Heart Failure eFigure 3. Analysis of Study Outcomes: MACE eFigure 4. Analysis of Study Outcomes: Rates of HHF and Emergency Department Visits for Patients With Heart Failure eFigure 5. Analysis of Study Outcomes: Rates of HHF and Emergency Department Visits for Patients With Cardiovascular Death eFigure 6. Analysis of Study Outcomes: Acute Myocardial Infarction eFigure 7. Analysis of Study Outcomes: All-Cause Mortality [file jamanetwopen-e2142078-s001.pdf]

Supplementary Online Content

Bhattarai M, Salih M, Regmi M, et al. Association of sodium-glucose cotransporter 2 inhibitors with cardiovascular outcomes in patients with type 2 diabetes and other risk factors for cardiovascular disease: a meta-analysis. *JAMA Netw Open*. 2022;5(1):e2142078. doi:10.1001/jamanetworkopen.2021.42078

eTable 1. Modified Jadad Scores of the Included Studies

eTable 2. Definition of Inclusion, Exclusion, Primary Outcome, and Secondary Outcome

eFigure 1. Funnel Plot

eFigure 2. Analysis of Study Outcomes: Cardiovascular Death or Hospitalization for Heart Failure

eFigure 3. Analysis of Study Outcomes: MACE

eFigure 4. Analysis of Study Outcomes: Rates of HHF and Emergency Department Visits for Patients With Heart Failure

eFigure 5. Analysis of Study Outcomes: Rates of HHF and Emergency Department Visits for Patients With Cardiovascular Death

eFigure 6. Analysis of Study Outcomes: Acute Myocardial Infarction

eFigure 7. Analysis of Study Outcomes: All-Cause Mortality

This supplementary material has been provided by the authors to give readers additional information about their work.

© 2022 Bhattarai M et al. *JAMA Network Open*.

eTable 1. Modified Jadad Scores of the Included Studies

Study ame	Was the research described as randomization?	Was the approach of randomization appropriate?	Was the research described as blinding?	Was the approach of blinding appropriate?	Was there a presentation of the withdrawals and dropouts?	Was there a presentation the inclusion/exclusion criteria?	Was the approach used to assess adverse effects described?	Was the approach of statistical analysis described?	total
CANVAS Neal et al	Yes (1)	Yes (1)	Yes Single (1)	Yes (1)	Yes (1)	Yes (1)	Yes (1)	Yes (1)	8
DAPA-HF McMurray et al	Yes (1)	Yes (1)	Yes Placebo Control (1)	Yes (1)	Yes (1)	Yes (1)	Yes (1)	Yes (1)	8
DECLARE-TIMI 58 Wiviott et al	Yes (1)	Yes (1)	Yes Single (1)	Yes (1)	Yes (1)	Yes (1)	Yes (1)	Yes (1	8
EMPA-REG OUTCOME Zinman et al	Yes (1)	Yes (1)	Yes Double (1)	Yes (1)	Yes (1)	Yes (1)	Yes (1)	Yes (1)	8
CREDENCE Perkovic et al	Yes (1)	Yes (1)	Yes Double (1)	Yes (1)	Yes (1)	Yes (1)	Yes (1)	Yes (1)	8
DAPA-CKD, Heerspink et al	Yes (1)	Yes (1)	Yes Double (1)	Yes (1)	Yes (1)	Yes (1)	Yes (1)	Yes (1)	8
EMPEROR-Reduced, Packer et al	Yes (1)	Yes (1)	Yes Double (1)	Yes (1)	Yes (1)	Yes (1)	Yes (1)	Yes (1)	8
VERTIS-CV, Cannon et al	Yes (1)	Yes (1)	Yes Double (1)	Yes (1)	Yes (1)	Yes (1)	Yes (1)	Yes (1)	8
SOLOIST-WHF, Bhatt et al	Yes (1)	Yes (1)	Yes Double (1)	Yes (1)	Yes (1)	Yes (1)	Yes (1)	Yes (1)	8
SCORED, Bhatt et al	Yes (1)	Yes (1)	Yes Double (1)	Yes (1)	Yes (1)	Yes (1)	Yes (1)	Yes (1)	8

© 2022 Bhattarai M et al. *JAMA Network Open*.

eTable 2. Definition of Inclusion, Exclusion, Primary Outcome, and Secondary Outcome

Study Name	Inclusion Criteria	Exclusion criteria	Primary outcome	Secondary outcome
EMPAREG-Outcome (Type 2 Diabetes) Zinman et al	Type 2 diabetes, adults (≥18) with BMI of 45 or less and an eGFR of at least 30 ml per minute per 1.73 m2 of BSA. All the patients had established cardiovascular disease and had received no glucose-lowering agents for at least 12 weeks before randomization and had a glycated hemoglobin level of at least 7.0% and no more than 9.0% or had received stable glucose-lowering therapy for at least 12 weeks before randomization and had a glycated hemoglobin level of at least 7.0% and no more than 10.0%.	Uncontrolled hyperglycemia with glucose >240 mg/dL after an overnight fast during placebo run-in and confirmed by a second measurement (not on the same day). Indication of liver disease, defined by serum levels of alanine aminotransferase, aspartate aminotransferase, or alkaline phosphatase above 3 x upper limit of normal during screening or run-in phase. Planned cardiac surgery or angioplasty within 3 months. Estimated glomerular filtration rate <30 ml/min/1.73 m2. Any uncontrolled endocrine disorder except type 2 diabetes	Death from cardiovascular causes, nonfatal myocardial infarction, or nonfatal stroke: primary outcome	The key secondary outcome was a composite of the primary outcome plus hospitalization for unstable angina.
CANVAS and CANVAS-R (Type 2 Diabetes), Neal et al	Type 2 diabetes (HgbA1c, ≥7.0% and ≤10.5%) and were either 30 years of age or older with a history of symptomatic atherosclerotic cardiovascular disease or 50 years of age or older with two or more of the following risk factors for cardiovascular disease: duration of diabetes of at least 10 years, systolic blood pressure higher than 140 mm Hg while they were receiving one or more antihypertensive agents, current smoking, microalbuminuria or macroalbuminuria, or HDL level of less than 1 mmol per liter (38.7 mg per deciliter). Participants were required to have eGFR at entry of more than 30 ml per minute per 1.73 m2 of BSA and to meet a range of other criteria.	History of diabetic ketoacidosis, type 1 diabetes, pancreas or beta-cell transplantation, or diabetes secondary to pancreatitis or pancreatectomy. H/o one or more severe hypoglycemic episode with in 6 months before screening, MI or unstable angina, revascularization procedure, or cerebrovascular accident with in 3 months before screening, or planned revascularization procedure, or history of NYHA IV cardiac disease	The primary outcome was a composite of death from cardiovascular causes, nonfatal myocardial infarction, or nonfatal stroke.	Secondary outcomes planned for sequential conditional hypothesis testing were death from any cause, death from cardiovascular causes, progression of albuminuria, and the composite of death from cardiovascular causes and hospitalization for heart failure
CREDENCE (type 2 DM and nephropathy) Perkovic et al	Eligible if they were at least 30 years of age and had type 2 diabetes, with HgbA1c of 6.5 to 12.0% (6.5 to 10.5% in Germany, according to a country amendment). They were also required to have chronic kidney disease, defined as an eGFR (GFR, as calculated by the Chronic Kidney Disease Epidemiology Collaboration formula) of 30 to <90 ml per minute per 1.73 m2 of BSA and albuminuria (urinary albumin-to-creatinine ratio, >300 to 5000, with albumin measured in milligrams and creatinine in grams), as	History of diabetic ketoacidosis or type 1 diabetes mellitus (T1DM).History of hereditary glucose-galactose malabsorption or primary renal glucosuria. Known medical history or clinical evidence suggesting nondiabetic renal disease. Renal disease that required treatment with immunosuppressive therapy or a history of chronic dialysis or renal transplant. Uncontrolled hypertension (systolic blood pressure [BP] ≥180 and/or diastolic BP ≥100 mmHg) by Week. Myocardial infarction, unstable angina, revascularization procedure (e.g., stent or bypass graft surgery), or	Composite of end- stage kidney disease (dialysis for at least 30 days, kidney transplant, or an estimated GFR of < 15 ml per 1.73 m2 sustained for at least 30 days according to central laboratory assessment), doubling of the serum creatinine level from baseline	Sequential hierarchical testing were specified in the following order: first, a composite of cardio-vascular death or hospitalization for heart failure; second, a composite of cardiovascular death, myocardial infarction, or stroke; third, hospitalization for heart failure; fourth, a composite of end-stage kidney disease, doubling of the serum creatinine

© 2022 Bhattarai M et al. *JAMA Network Open*.

	measured in a central laboratory. There was a prespecified plan to include approximately 60% of patients with an estimated GFR of 30 to <60 ml per minute per 1.73 m2.	cerebrovascular accident within 12 weeks before randomization, or a revascularization procedure is planned during the trial.<br>Current or history of heart failure of New York Heart Association (NYHA) class IV cardiac disease (The Criteria Committee of the NYHA).	((average of randomization and prerandomization value) sustained for at least 30 days according to central laboratory assessment, or death from renal or cardiovascular disease.	level, or renal death; fifth, cardiovascular death; sixth, death from any cause; and seventh, a composite of cardiovascular death, myocardial infarction, stroke, or hospitalization for heart failure or for unstable angina. All other efficacy outcomes were exploratory.
DAPA-HF (HFrEF) McMurray et al	Eligibility requirements included an age at least 18 years, LVEF of 40% or less, and NYHA class II, III, or IV symptoms. Patients were required to have a plasma level of N-terminal pro–B-type natriuretic peptide (NT-proBNP) of at least 600 pg per milliliter (or ≥400 pg per milliliter if they had been hospitalized for heart failure within the previous 12 months). Patients with atrial fibrillation or atrial flutter on baseline electrocardiography were required to have an NT-proBNP level of at least 900 pg per milliliter, regardless of their history of hospitalization for heart failure. Patients were required to receive standard heart failure device therapy (an ICD, CRT, or both) and standard drug therapy.	Recent treatment with or unacceptable side effects associated with an SGLT2 inhibitor<br>Type 1 diabetes mellitus<br>Symptoms of hypotension or SBP < 95 mm Hg<br>eGFR < 30 ml per minute per 1.73 m2 BSA (or rapidly declining renal function)	The primary outcome was a composite of worsening heart failure or death from cardiovascular causes. An episode of worsening heart failure was either an unplanned hospitalization or an urgent visit resulting in intravenous therapy for heart failure.	A key secondary outcome was a composite of hospitalization for heart failure or cardiovascular death. The additional secondary outcomes were the total number of hospitalizations for heart failure (including repeat admissions) and cardiovascular deaths; the change from baseline to 8 months in the total symptom score on the Kansas City Cardiomyopathy Questionnaire
DECLARE - TIMI 58 (Type 2 diabetes) Wiviott et al	Eligible patients were 40 years of age or older and had type 2 diabetes, HgbA1c of at least 6.5% but less than 12.0%, and a creatinine clearance of 60 ml or more per minute. Eligible patients also had multiple risk factors for atherosclerotic cardiovascular disease or had established atherosclerotic cardiovascular disease (defined as clinically evident ischemic heart disease, ischemic cerebrovascular disease, or peripheral artery disease). Participants with multiple risk factors were men 55 years of age or older or women 60 years of age or older who had one or more traditional risk factors, including hypertension, dyslipidemia (defined as a low-density lipoprotein cholesterol level >130 mg per	Diagnosis of type 1 DM<br>History of bladder cancer or history of radiation therapy to the lower abdomen or pelvis at any time<br>Chronic cystitis and/or recurrent urinary tract infections<br>Pregnant or breast-feeding patients	The primary safety outcome was a composite of major adverse cardiovascular events (MACE), defined as cardiovascular death, myocardial infarction, or ischemic stroke. The primary efficacy outcomes were MACE and a composite of cardiovascular death or hospitalization for heart failure.	Secondary outcomes 1. renal composite outcome, defined as a sustained decrease of 40% or more in estimated glomerular filtration rate (eGFR) — calculated by means of the Chronic Kidney Disease Epidemiology Collaboration equation22 to less than 60 ml per minute per 1.73 m2 of body-surface area, new end-stage renal disease, or death from renal or cardiovascular causes. 2. Death from any cause

© 2022 Bhattarai M et al. *JAMA Network Open*.

	deciliter [3.36 mmol per liter] or the use of lipid-lowering therapies), or use of tobacco.			
DAPA-CKD (CKD+- DM) Main outcome renal,2 outcomes interest, Heerspink et al	With or without type 2 diabetes who had eGFR of 25 to 75 ml per minute per 1.73 m2 of BSA and a urinary albumin-to-creatinine ratio (with albumin measured in milligrams and creatinine measured in grams) of 200 to 5000 were eligible for participation. All the participants were required to be receiving a stable dose of an ACE inhibitor or ARB for at least 4 weeks before screening. However, participants who were documented to be unable to take ACE inhibitors or ARBs were allowed to participate. Key exclusion criteria were a documented diagnosis of type 1 diabetes, polycystic kidney disease, lupus nephritis, or antineutrophil cytoplasmic antibody associated vasculitis.	Key exclusion criteria were a documented diagnosis of type 1 diabetes, polycystic kidney disease, lupus nephritis, or antineutrophil cytoplasmic antibody–associated vasculitis. Participants who had received immunotherapy for primary or secondary kidney disease within 6 months before enrollment were also excluded.	The primary outcome was a composite of a sustained decline in the estimated GFR of at least 50%, end-stage kidney disease, or death from renal or cardiovascular causes.	Secondary outcomes (also assessed in time-to-event analyses) were, in hierarchical order, the composite kidney outcome of a sustained decline in the estimated GFR of at least 50%, end stage kidney disease, or death from renal causes; a composite cardiovascular outcome defined as hospitalization for heart failure or death from cardiovascular causes; and death from any cause.

© 2022 Bhattarai M et al. *JAMA Network Open*.

EMPEROR-Reduced, Packer et al	Adults (≥18 years of age) who had chronic heart failure (functional class II, III, or IV) with LVEF of 40% or less. All the patients were receiving appropriate treatments for heart failure, including diuretics, inhibitors of RAS and neprilysin, beta-blockers, mineralocorticoid receptor antagonists, and, when indicated, cardiac devices.	-Myocardial infarction, coronary artery bypass graft surgery, or other major cardiovascular surgery, stroke or TIA (Transient Ischemic Attack) in past 90 days prior to Visit 1<br>-Heart transplant recipient, or listed for heart transplant<br>-Acute decompensated HF<br>-Systolic blood pressure (SBP) >= 180 mmHg at Visit 2.<br>-Symptomatic hypotension and/or a SBP < 100 mmHg<br>-Indication of liver disease<br>-Impaired renal function, defined as eGFR (Estimated Glomerular Filtration Rate) < 20 mL/min/1.73 m2 (CKD-EPI (Chronic Kidney Disease - Epidemiology Collaboration Equation)) or requiring dialysis<br>-History of ketoacidosis<br>-Current use or prior use of a SGLT (Sodium-glucose co-transporter)-2 inhibitor or combined SGLT-1 and 2 inhibitor<br>-Currently enrolled in another investigational device or drug study<br>-Known allergy or hypersensitivity to empagliflozin or other SGLT-2 inhibitors<br>-Women who are pregnant, nursing, or who plan to become pregnant while in the trial	The primary outcome was a composite of cardiovascular death or hospitalization for worsening heart failure.	The first secondary outcome was the occurrence of all adjudicated hospitalizations for heart failure, including first and re-current events. The second secondary outcome was the rate of the decline in the estimated GFR during double-blind treatment.
VERTIS-CV, Cannon et al	Eligible if they were at least 40 years of age and had type 2 diabetes (with HgbA1c of 7.0 to 10.5%) and established atherosclerotic cardiovascular disease involving the coronary, cerebrovascular, or peripheral arterial systems.	Key exclusion criteria were a history of type 1 diabetes or ketoacidosis and an estimated glomerular filtration rate below 30 ml per minute per 1.73 m2 of body-surface area.	Death from cardiovascular causes, nonfatal myocardial infarction, or nonfatal stroke	The key secondary outcomes, assessed in time-to-event analyses and in a hierarchical statistical testing sequence, were a composite of death from cardiovascular causes or hospitalization for heart failure; death from cardiovascular causes; and a composite of death from renal causes, renal replacement therapy, or doubling of the serum creatinine level.

© 2022 Bhattarai M et al. *JAMA Network Open*.

SOLOIST-WHF, Bhatt et al	eligible for enrollment in the trial if they were 18 to 85 years of age and had been hospitalized because of the presence of signs and symptoms of heart failure and received treatment with intravenous diuretic therapy. Patients were also required to have received a previous diagnosis of type 2 diabetes before the index admission or to have laboratory evidence to support a diagnosis of type 2 diabetes during the index admission. Exclusion criteria included end-stage heart failure or recent acute coronary syndrome, stroke, percutaneous coronary intervention or coronary-artery bypass surgery, or an estimated GFR of less than 30 ml per minute per 1.73 m2 of body surface area.	Exclusion criteria included end-stage heart failure or recent acute coronary syndrome, stroke, percutaneous coronary intervention or coronary artery bypass surgery, or an estimated GFR of less than 30 ml per minute per 1.73 m2 of body surface area.	Deaths from cardiovascular causes and hospitalizations and urgent visits for heart failure)	The revised secondary endpoints were the total number of hospitalizations and urgent vis-<br>its for heart failure; the incidence of death from cardiovascular causes; the incidence of death from any cause; the total number of deaths from cardiovascular causes, hospitalizations for heart failure, nonfatal myocardial infarctions, and nonfatal strokes; the total number of deaths from cardiovascular causes, hospitalizations and urgent visits for heart failure, and events of heart failure during hospitalization; the change in score on the Kansas City Cardiomyopathy Questionnaire–12 item (KCCQ-12; scores range from 0 to 100, with higher scores indicating better quality of life) to month 4; and the change in the estimated GFR.31
SCORED, Bhatt et al	Persons 18 years of age or older with type 2 diabetes mellitus with a glycated hemoglobin level of 7% or higher, chronic kidney disease (eGFR, 25 to 60 ml per minute per 1.73 m2 of body-surface area), and additional cardiovascular risk factors were enrolled. The risk factors consisted of at least one major cardiovascular risk factor in those18 years of age or older or at least two minor cardiovascular risk factors in those 55 years of age or older. An exclusion criterion was any plan to start an SGLT2 inhibitor during the trial.	-Antihyperglycemic treatment has not been stable within 12 weeks prior to screening.<br>-Planned coronary procedure or surgery after randomization.<br>-Lower extremity complications (such as skin ulcer, infection, osteomyelitis, and gangrene) identified during screening and requiring treatment at randomization.<br>-Planning to start a sodium-glucose linked transporter-2 (SGLT2) inhibitor during the study.	The primary endpoint was changed during the trial to the composite of the total number of deaths from cardiovascular causes, hospitalizations for heart failure, and urgent visits for heart failure.	-Total no. or hospitalizations for HF and urgent visits for HF Deaths from cardiovascular causes Total no. of deaths from cardiovascular causes, hospitalizations for HF, nonfatal myocardial infarctions, and nonfatal strokes<br>-Total no. of deaths from cardiovascular causes, hospitalizations for HF, urgent visits for HF, and events of HF during hospitalization<br>-First occurrence of a sustained

© 2022 Bhattarai M et al. *JAMA Network Open*.

				decrease of ≥50% in the eGFR from baseline for ≥30 days, long-term dialysis, renal transplantation, or sustained eGFR of <15 ml/min/1.73 m2 for ≥30 days<br>-Deaths from any cause Total no. of deaths from cardiovascular causes, nonfatal myocardial infarctions, and nonfatal strokes

© 2022 Bhattarai M et al. *JAMA Network Open*.

eFigure 1. Funnel Plot

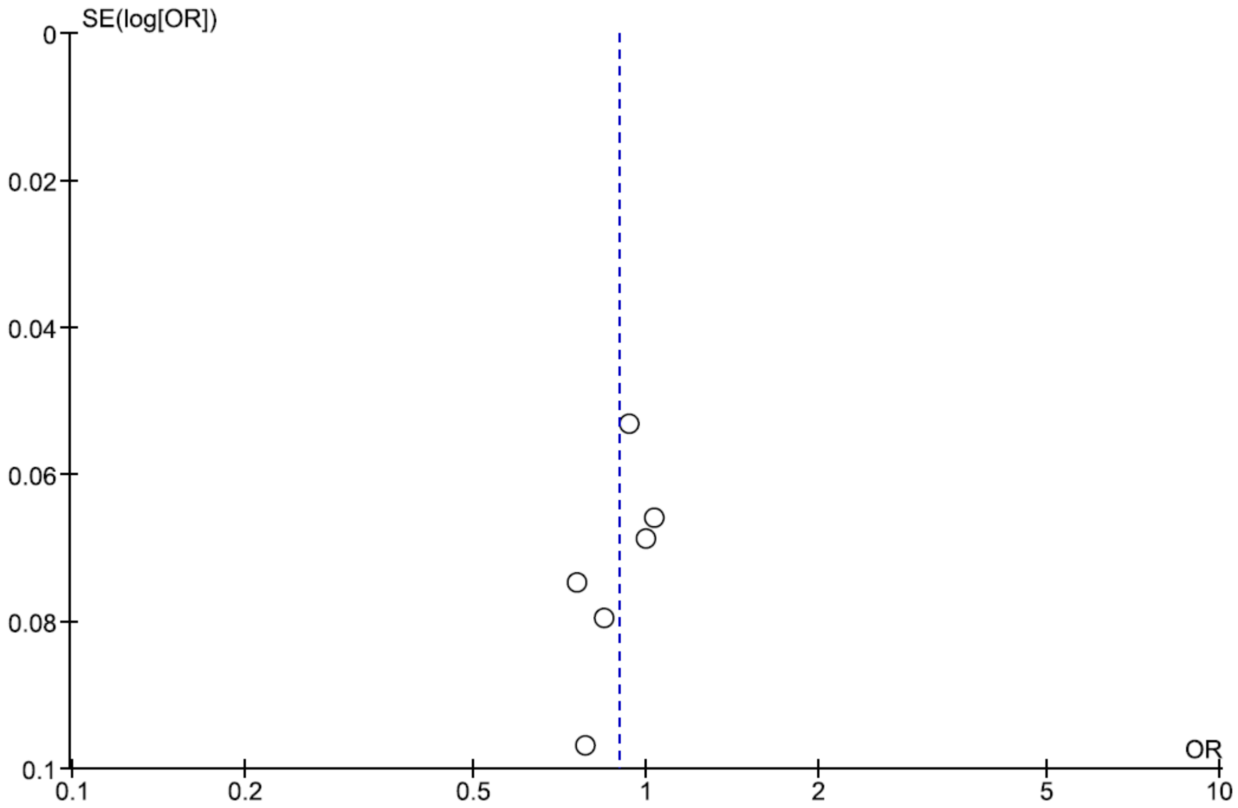

© 2022 Bhattarai M et al. *JAMA Network Open*.

eFigure 2. Analysis of Study Outcomes: Cardiovascular Death or Hospitalization for Heart Failure

	SGLT2-I		Placebo			Odds Ratio	
Study or Subgroup	Events	Total	Events	Total	Weight	M-H, Random, 95% CI	Year
EMPA-REG OUTCOME	265	4687	198	2333	9.9%	0.65 [0.53, 0.78]	2015
CANVAS and CANVAS-R	368	5795	292	4347	10.2%	0.94 [0.80, 1.10]	2017
CREDENCE	179	2202	253	2199	9.8%	0.68 [0.56, 0.83]	2019
DECLARE-TIMI 58	417	8582	496	8578	10.4%	0.83 [0.73, 0.95]	2019
DAPA-HF	386	2373	502	2371	10.3%	0.72 [0.62, 0.84]	2019
DAPA-CKD	100	2152	138	2152	9.0%	0.71 [0.55, 0.93]	2020
SCORED	400	5292	530	5292	10.4%	0.73 [0.64, 0.84]	2020
SOLOIST-WHF	245	608	355	614	9.5%	0.49 [0.39, 0.62]	2020
EMPEROR-Reduced	361	1863	462	1867	10.2%	0.73 [0.63, 0.85]	2020
VERTIS-CV	444	5499	530	2747	10.4%	0.37 [0.32, 0.42]	2020
**Total (95% CI)**		**39053**		**32500**	**100.0%**	**0.67 [0.55, 0.80]**	
Total events	3165		3756				

Heterogeneity: Tau² = 0.08; Chi² = 114.50, df = 9 (P < 0.00001); I² = 92%

Test for overall effect: Z = 4.26 (P < 0.0001)

Odds Ratio
M-H, Random, 95% CI

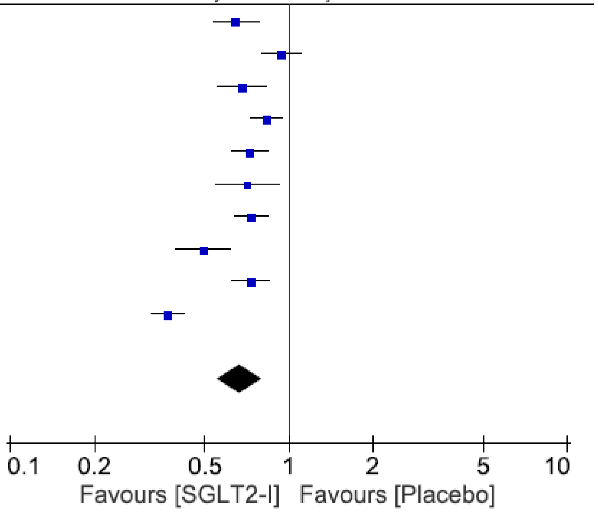

© 2022 Bhattarai M et al. *JAMA Network Open*.

eFigure 3. Analysis of Study Outcomes: MACE

	SGLT2-I		Placebo			Odds Ratio	
**Study or Subgroup**	**Events**	**Total**	**Events**	**Total**	**Weight**	**M-H, Random, 95% CI**	**Year**
EMPA-REG OUTCOME	490	4687	282	2333	15.6%	0.85 [0.73, 0.99]	2015
CANVAS and CANVAS-R	608	5795	442	4347	17.8%	1.04 [0.91, 1.18]	2017
DECLARE-TIMI 58	756	8582	803	8578	19.9%	0.94 [0.84, 1.04]	2019
CREDENCE	217	2202	269	2199	13.1%	0.78 [0.65, 0.95]	2019
VERTIS-CV	735	5499	368	2747	17.3%	1.00 [0.87, 1.14]	2020
SCORED	343	5292	442	5292	16.3%	0.76 [0.66, 0.88]	2020
**Total (95% CI)**		**32057**		**25496**	**100.0%**	**0.90 [0.81, 0.99]**	
Total events	3149		2606				

Heterogeneity: Tau² = 0.01; Chi² = 14.78, df = 5 (P = 0.01); I² = 66%

Test for overall effect: Z = 2.19 (P = 0.03)

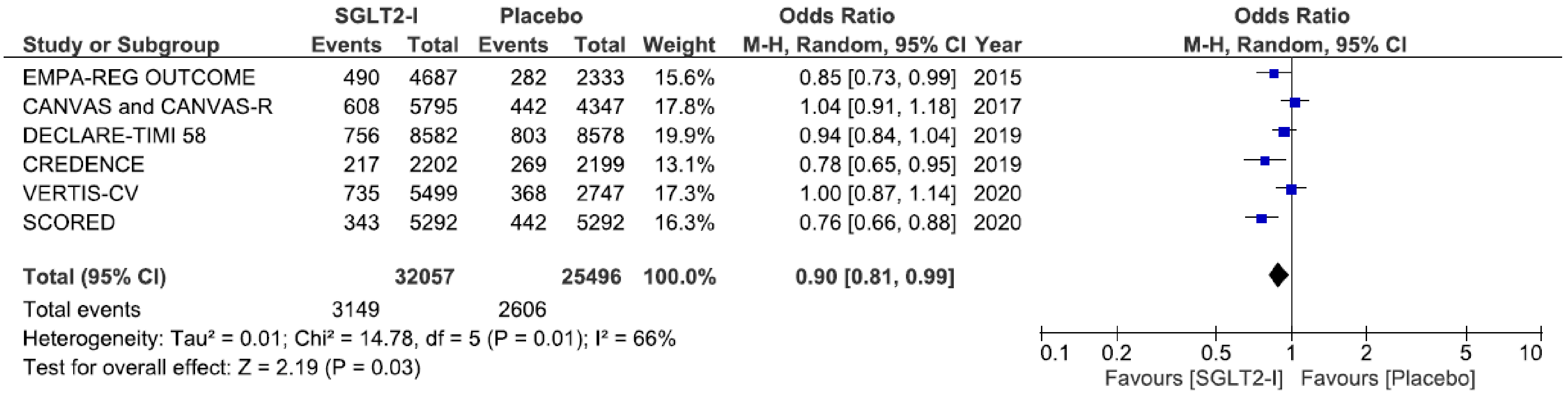

© 2022 Bhattarai M et al. *JAMA Network Open*.

eFigure 4. Analysis of Study Outcomes: Rates of HHF and Emergency Department Visits for Patients With Heart Failure

	SGLT2-I		Placebo			Odds Ratio	
**Study or Subgroup**	**Events**	**Total**	**Events**	**Total**	**Weight**	**M-H, Random, 95% CI**	**Year**
EMPA-REG OUTCOME	126	4687	95	2333	6.9%	0.65 [0.50, 0.85]	2015
CANVAS and CANVAS-R	124	5795	122	4347	7.9%	0.76 [0.59, 0.98]	2017
CREDENCE	89	2202	141	2199	6.9%	0.61 [0.47, 0.81]	2019
DECLARE-TIMI 58	212	8582	286	8578	14.7%	0.73 [0.61, 0.88]	2019
DAPA-HF	237	2373	326	2371	15.1%	0.70 [0.58, 0.83]	2019
EMPEROR-Reduced	246	1863	342	1867	15.1%	0.68 [0.57, 0.81]	2020
SOLOIST-WHF	194	608	297	614	9.2%	0.50 [0.40, 0.63]	2020
SCORED	245	5292	360	5292	16.9%	0.67 [0.56, 0.79]	2020
VERTIS-CV	139	5499	99	2747	7.4%	0.69 [0.53, 0.90]	2020
**Total (95% CI)**		**36901**		**30348**	**100.0%**	**0.67 [0.62, 0.72]**	
Total events	1612		2068				

Heterogeneity: Tau² = 0.00; Chi² = 8.65, df = 8 (P = 0.37); I² = 8%

Test for overall effect: Z = 10.83 (P < 0.00001)

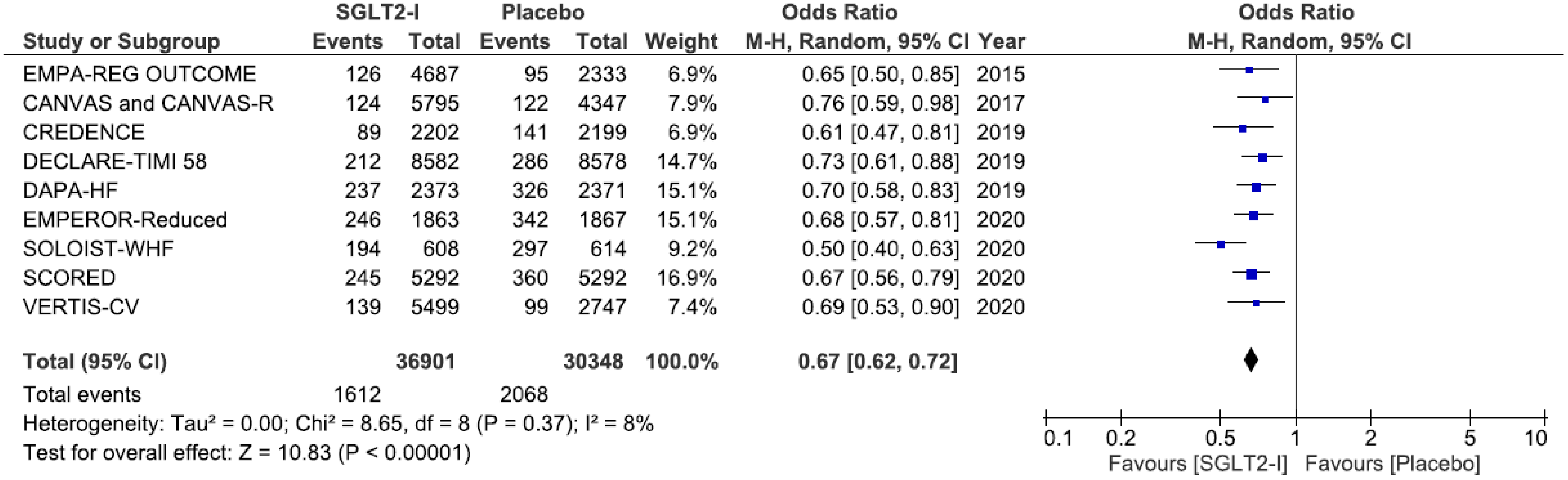

© 2022 Bhattarai M et al. *JAMA Network Open*.

eFigure 5. Analysis of Study Outcomes: Rates of HHF and Emergency Department Visits for Patients With Cardiovascular Death

	SGLT2-I		Placebo			Odds Ratio	
**Study or Subgroup**	**Events**	**Total**	**Events**	**Total**	**Weight**	**M-H, Random, 95% CI**	**Year**
EMPA-REG OUTCOME	172	4687	137	2333	10.0%	0.61 [0.49, 0.77]	2015
CANVAS and CANVAS-R	262	5795	179	4347	11.7%	1.10 [0.91, 1.34]	2017
DECLARE-TIMI 58	245	8582	249	8578	12.5%	0.98 [0.82, 1.18]	2019
CREDENCE	110	2202	140	2199	8.9%	0.77 [0.60, 1.00]	2019
DAPA-HF	227	2373	273	2371	12.1%	0.81 [0.67, 0.98]	2019
VERTIS-CV	341	5499	184	2747	12.1%	0.92 [0.77, 1.11]	2020
SOLOIST-WHF	51	608	58	614	5.1%	0.88 [0.59, 1.30]	2020
SCORED	155	5292	170	5292	10.4%	0.91 [0.73, 1.13]	2020
EMPEROR-Reduced	187	1863	202	1867	10.9%	0.92 [0.75, 1.13]	2020
DAPA-CKD	65	2152	80	2152	6.5%	0.81 [0.58, 1.13]	2020
**Total (95% CI)**		**39053**		**32500**	**100.0%**	**0.87 [0.79, 0.97]**	
Total events	1815		1672				

Heterogeneity: Tau² = 0.01; Chi² = 18.74, df = 9 (P = 0.03); I² = 52%

Test for overall effect: Z = 2.62 (P = 0.009)

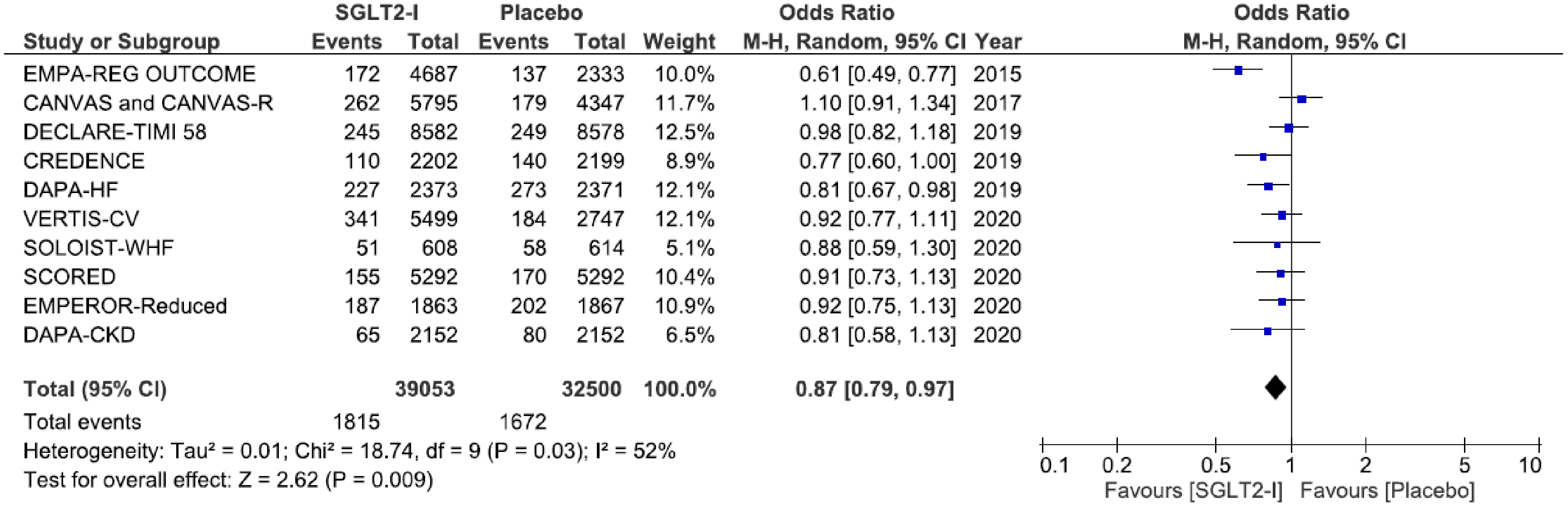

© 2022 Bhattarai M et al. *JAMA Network Open*.

eFigure 6. Analysis of Study Outcomes: Acute Myocardial Infarction

	SGLT2-I		Placebo			Odds Ratio	
**Study or Subgroup**	**Events**	**Total**	**Events**	**Total**	**Weight**	**M-H, Random, 95% CI**	**Year**
EMPA-REG OUTCOME	261	4687	141	2333	17.2%	0.92 [0.74, 1.13]	2015
CANVAS and CANVAS-R	219	5795	163	4347	18.0%	1.01 [0.82, 1.24]	2017
DAPA-HF	53	2368	55	2368	5.3%	0.96 [0.66, 1.41]	2019
DECLARE-TIMI 58	393	8582	441	8578	39.5%	0.89 [0.77, 1.02]	2019
VERTIS-CV	330	5499	158	2747	20.1%	1.05 [0.86, 1.27]	2020
**Total (95% CI)**		**26931**		**20373**	**100.0%**	**0.95 [0.87, 1.03]**	
Total events	1256		958				

Heterogeneity: Tau² = 0.00; Chi² = 2.34, df = 4 (P = 0.67); I² = 0%

Test for overall effect: Z = 1.22 (P = 0.22)

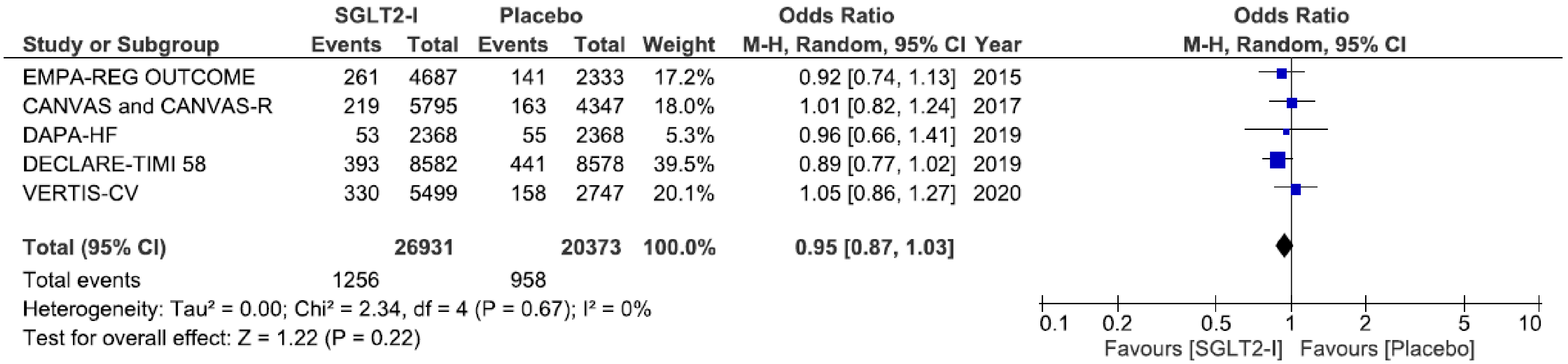

© 2022 Bhattarai M et al. *JAMA Network Open*.

eFigure 7. Analysis of Study Outcomes: All-Cause Mortality

	SGLT2-I		Placebo			Odds Ratio	
Study or Subgroup	Events	Total	Events	Total	Weight	M-H, Random, 95% CI	Year
EMPA-REG OUTCOME	269	4687	194	2333	10.0%	0.67 [0.55, 0.81]	2015
CANVAS and CANVAS-R	391	5795	274	4347	11.6%	1.08 [0.92, 1.26]	2017
DAPA-HF	276	2373	329	2371	11.0%	0.82 [0.69, 0.97]	2019
DECLARE-TIMI 58	529	8582	570	8578	13.6%	0.92 [0.82, 1.04]	2019
CREDENCE	168	2202	201	2199	9.1%	0.82 [0.66, 1.02]	2019
SOLOIST-WHF	65	608	76	614	4.9%	0.85 [0.60, 1.20]	2020
VERTIS-CV	473	5499	254	2747	11.6%	0.92 [0.79, 1.08]	2020
DAPA-CKD	101	2152	146	2152	7.3%	0.68 [0.52, 0.88]	2020
EMPEROR-Reduced	249	1863	266	1867	10.3%	0.93 [0.77, 1.12]	2020
SCORED	246	5292	246	5292	10.5%	1.00 [0.83, 1.20]	2020
**Total (95% CI)**		**39053**		**32500**	**100.0%**	**0.87 [0.80, 0.96]**	
Total events	2767		2556				

Heterogeneity: Tau² = 0.01; Chi² = 21.87, df = 9 (P = 0.009); I² = 59%
Test for overall effect: Z = 2.91 (P = 0.004)

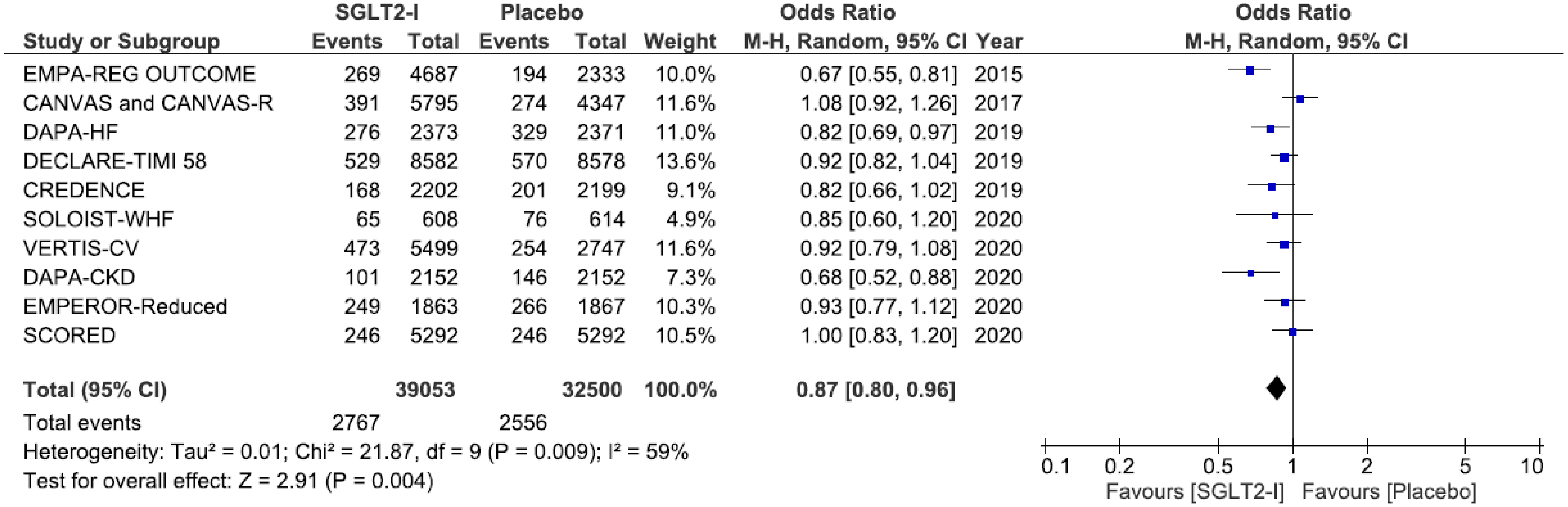

© 2022 Bhattarai M et al. *JAMA Network Open*.
